# Segment-specific generation of *Drosophila* Capability neuropeptide neurons by multi-faceted Hox cues

**DOI:** 10.1016/j.ydbio.2011.02.015

**Published:** 2011-05-01

**Authors:** Anke Suska, Irene Miguel-Aliaga, Stefan Thor

**Affiliations:** a Department of Clinical and Experimental Medicine, Linkoping University, Linkoping, SE-58185, Sweden; b University of Cambridge, Cambridge, CB2 3EJ, UK

**Keywords:** Apoptosis, Hox genes, Neuropeptide neurons, RHG genes

## Abstract

In the *Drosophila* ventral nerve cord, the three pairs of Capability neuropeptide-expressing Va neurons are exclusively found in the second, third and fourth abdominal segments (A2–A4). To address the underlying mechanisms behind such segment-specific cell specification, we followed the developmental specification of these neurons. We find that Va neurons are initially generated in all ventral nerve cord segments and progress along a common differentiation path. However, their terminal differentiation only manifests itself in A2–A4, due to two distinct mechanisms: segment-specific programmed cell death (PCD) in posterior segments, and differentiation to an alternative identity in segments anterior to A2. Genetic analyses reveal that the Hox homeotic genes are involved in the segment-specific appearance of Va neurons. In posterior segments, the Hox gene *Abdominal-B* exerts a pro-apoptotic role on Va neurons, which involves the function of several RHG genes. Strikingly, this role of *Abd-B* is completely opposite to its role in the segment-specific apoptosis of other classes of neuropeptide neurons, the dMP2 and MP1 neurons, where *Abd-B* acts in an anti-apoptotic manner. In segments A2–A4 we find that *abdominal A* is important for the terminal differentiation of Va cell fate. In the A1 segment, *Ultrabithorax* acts to specify an alternate Va neuron fate. In contrast, in thoracic segments, *Antennapedia* suppresses the Va cell fate. Thus, Hox genes act in a multi-faceted manner to control the segment-specific appearance of the Va neuropeptide neurons in the ventral nerve cord.

## Research Highlights

► The nervous system contains many unique segment-specific neurons. ► The Drosophila the Capability neuropeptide-expressing Va neurons are only found in a few segments. ► The Va neurons are generated in all segment, but are removed by cell death in some segments. ► In other segments, Va neurons differentiate into different neuronal sub-types. ► These events are controlled by an interplay of Hox genes and cell death genes of the RHG family.

## Introduction

A common feature of most nervous systems is the appearance of unique neuronal subtypes only within certain segments. Studies have shown that this may result from several developmental mechanisms, the first of which is the segment-specific generation of unique sets of progenitor cells—for example, certain segments of the *Drosophila* developing CNS (tritocerebrum (B3) and suboesophageal 1 (S1)) appear to contain fewer progenitor cells (neuroblasts) (Urbach and Technau, 2004). Second, segment-specific lineage size control has been described, such that equivalent neuroblasts may generate different sized lineages in different segments (Schmid et al., 1999; Schmidt et al., 1997). This may be due either to differences in lineage progression (by apoptosis or cell cycle exit) or to early changes in asymmetric cell division by segment-specific control of genes affecting progenitor behavior (Berger et al., 2005a). Third, once generated, segment-specific events may dictate distinct terminal cell fates in different segments (Karlsson et al., 2010). Finally, neurons may be generated throughout the neuro-axes, and be similarly specified, but may be removed in a segment-specific manner by subsequent apoptosis (Miguel-Aliaga and Thor, 2004; Rogulja-Ortmann et al., 2008). The Hox homeotic genes have been found to be involved in several of these events, but our understanding of Hox gene involvement in these processes is still rudimentary.

The *Drosophila* ventral nerve cord (VNC) contains an estimated 10,000 cells, the majority of which are neurons. Within the neuronal subset, only 200 cells specifically express neuropeptides and are often referred to as being peptidergic (Miguel-Aliaga et al., 2004; Park et al., 2008). Each peptidergic subclass typically expresses only one out of a total of some 30 neuropeptide genes (Park et al., 2008). Intriguingly, although the VNC consists of repetitive segments (neuromeres), often containing similar sets of neurons and glia, peptidergic neurons display striking segment specificity (Miguel-Aliaga et al., 2004; Park et al., 2008). Undoubtedly, this segment specificity plays important roles to control segment- and region-specific physiological output. But in addition, and most importantly for this study, the segment-specific appearance of peptidergic neurons, together with their robust expression of different neuropeptide genes, makes them excellent “model neurons” for addressing segment-specific neuronal subtype generation and differentiation. We have previously capitalized upon this notion and studied several segment-specific peptidergic neurons, such as the A6–A8-specific dMP2 and MP1 neurons (Miguel-Aliaga and Thor, 2004), as well as the thoracic-specific Nplp1 and FMRFamide neurons (Karlsson et al., 2010). These studies have identified segment-specific lineage truncation, cell fate specification, as well as apoptosis as underlying mechanisms. In all three mechanisms, homeotic genes of the Hox family were found to play critical roles.

Here, we address the segment-specific appearance of another class of peptidergic neurons, the Capability (Capa) expressing Va neurons (O'Brien and Taghert, 1998). Using a number of markers, we find that these neurons are initially generated in all VNC segments, and partially differentiate, as evident by expression of several cell-fate determinants including Capa itself. By stage 16, Va neurons in all abdominal segments posterior to A4 undergo apoptosis, while the A1–A4 Va neurons survive into larval stages. However, from these four segments, only A2–A4 contains Va neurons which express Capa. Similar to dMP2 and MP1, the RHG genes are also involved in Va neuron PCD. In addition, we find that Va PCD is under the control of *Abdominal-B*, but in contrast to its function in the related peptidergic dMP2 and MP1 neurons, in Va neurons it acts in a pro-apoptotic role. These findings reveal striking differences in Hox gene function with respect to PCD in related neurons and provide a platform for addressing context-dependent Hox function with respect to PCD. The other Bithorax-complex Hox genes, *abdominal A* and *Ultrabithorax*, in turn are important for the differentiation of Va neurons. In contrast, the *Antennapedia* Hox gene suppresses Va neuron differentiation in thoracic segments. Thus, Hox genes act in several ways to ensure the segment-specific appearance of a distinct set of neurons.

## Materials and methods

### Fly stocks

The following fly stocks were used: *abd-A* mutants*: abd-A*^*P10*^, *abd-A*^*MXI*^. *Abd-Bm* mutants: *Abd-B*^*M1*^, *Abd-B*^*M2*^ (Sanchez-Herrero et al., 1985). *Antp* mutants: *Antp*^*12*^, *Antp*^*14*^. *Ubx* mutants: *Ubx*^*9.22*^, *Ubx*^*9.27*^; *UAS-Antp*, *UAS-Ubx*, *UAS-abd-A* (obtained from F. Hirth); *UAS-Abd-Bm* (obtained from J. Castelli-Gair); *UAS-Apoliner* (obtained from P.-L. Bardet); *dac-Gal4* (obtained from G. Mardon); *H99*, *XR38*, *X14* and *X25* deficiencies (obtained from K. White); *elav*^*GAL4*^ (*elavC155*). *w*^*1118*^ was used as a wild-type strain. Mutations were maintained over standard balancers with *GFP* markers. Mutants were identified by the absence of *GFP* expression.

### Immunohistochemistry

Immunohistochemistry was carried out as previously described (Allan et al., 2003). Rabbit α-proCapa was generated by injecting rabbits (Agrisera AB, Umea, Sweden) with a synthetic peptide (CKRSVDAKSFADISKGQKELN) corresponding to the C-terminal part of proCapa (Flybase). Antibodies were affinity-purified, pre-absorbed against early embryos, and used at 1:1000. Other antibodies used were mouse α-Dac dac2-3 (1:25) (Developmental Studies Hybridoma Bank), rabbit α-caspase-3 mAb (1:50) (Cell Signaling Technology), guinea pig α-Dimm (1:500) (Baumgardt et al., 2007), mAb α-Ubx (FP3.38; 1:10) (provided by R. White), mAb α-Abd-A (1:400) (provided by I. Duncan); mAb α-Antp (1:10) and mAb α-Abd-B (1:10) (Developmental Studies Hybridoma Bank, Iowa City, IA, US). FITC-, Rhodamine-Red-X- and Cy5-conjugated secondary antibodies were obtained from Jackson Immunolabs and used at 1:200 (1:100 for the Cy5-conjugated antibody).

### Confocal imaging and data acquisition

Zeiss LSM 5 or Zeiss META 510 Confocal microscopes were used to collect data for all fluorescent images; confocal stacks were merged using LSM software or Corel Paint Shop Pro Photo X2 (Ottawa, Canada). Statistical analysis was performed using Microsoft Excel.

## Results

### Developmental appearance of the peptidergic, Capability-expressing Va neurons

**Fig. 1:**
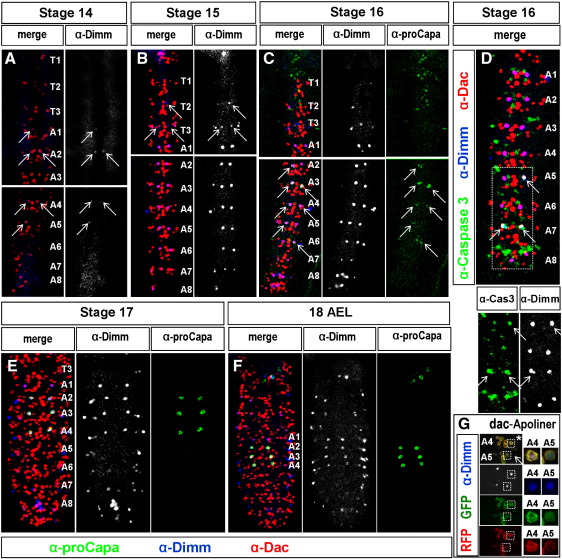
Developmental appearance of the Capa-expressing Va neurons. (A) Va neurons are first apparent at stage 14, when anterior abdominal Va neurons begin to express the transcription factors Dac and Dimm (arrows). (B) At stage 15, a pair of Dac/Dimm-positive Va neurons is present in nearly every abdominal segment of the VNC, but expression levels decrease towards posterior segments, and in A8 no Dac/Dimm expression can be observed. In thoracic segments (arrows) Dimm expression marks the existence of Va neurons, but Dac co-expression is missing. (C) Capa expression commences at stage 16 (arrows) in five abdominal segments (A2 to A6), and expression of Dac/Dimm is observed in all abdominal segments, including A8. Weak Dimm expression is found in T3 but is gradually lost completely in more anterior thoracic segments. (D) Simultaneously with the onset of Capa expression, caspase-3 appears in posterior Va neurons (arrows) indicating the beginning of apoptosis. (E) and (F) At stage 17 and 18 AEL only four pairs of Va neurons remain (A1–A4), three of which express proCapa (A2–A4). The remaining one is defined by the co-expression of the transcription factors Dac and Dimm. (G) Expression of the Apoliner PCD reporter reveals the death of Va neurons in A5 (arrow) when compared to the surviving Va neurons in A4 (star). In living cells, eGFP is tethered to mRFP and resides at the cellular membrane (star). In dying cells, caspase-mediated cleavage releases eGFP, which localizes to the nucleus.

In the *Drosophila* larval VNC, a set of six peptidergic neurons expresses the Capa neuropeptides, encoded by the *capability* gene (Kean et al., 2002). In the larvae, these neurons are present only in three of the abdominal segments (A2–A4), located at the ventral surface of the nerve cord; hence they have been named ventral–abdominal (Va) neurons (O'Brien and Taghert, 1998). They can be identified already at late embryonic stages of *Drosophila* development (Fig. 1F), but their developmental appearance was hitherto not investigated in detail. To address the segment-specific generation of Va neurons, we examined the expression of Capa throughout embryonic development. Staining for proCapa revealed that peptidergic Va neurons are apparent already at stage 16. Surprisingly, at this stage, proCapa expression was not confined to segments A2, A3 and A4 (Fig. 1C). By embryonic stage 17, however, the expression of Capa was restricted to the three pairs of neurons in A2–A4 (Fig. 1E–F), similar to that observed at larval stages (not shown; Kean et al., 2002). In addition to the six Va neurons present in the VNC, a pair of Capa expressing neurons are also found in the S1 segment (Fig. 1F; Kean et al., 2002). However in this study we will address the generation of Va neurons in the VNC i.e. segments T1–T3 and A1–A9.

The bHLH transcription factor Dimmed (Dimm) is selectively expressed in the majority of peptidergic neurons (Baumgardt et al., 2007; Hewes et al., 2003; Miguel-Aliaga et al., 2004; Park et al., 2004; Park et al., 2008) and is important for the high-level neuropetide expression and high-level secretory cellular properties (Baumgardt et al., 2007; Hamanaka et al., 2011; Hewes et al., 2003; Miguel-Aliaga et al., 2004; Park et al., 2004). As anticipated, Dimm was found to be expressed also in Capa-positive neurons. Additionally, the transcriptional co-factor Dachshund (Dac) is expressed in the Va cells (Miguel-Aliaga et al., 2004). Thus, Dimm/Dac co-expression will exclusively label the Va neurons and will label these neurons prior to Capa neuropeptide expression. Using these markers, we can first observe Va neurons in mid-abdominal segments at stage 14 (Fig. 1A), and from there we observe them in progression both anteriorly and posteriorly. Interestingly, at stage 15, Dimm/Dac-positive cells were present almost in every abdominal segment, although the expression gradually weakened towards the posterior end (Fig. 1B). In thoracic segments, expression of Dimm in the same position as the abdominal Dimm/Dac cells suggested the presence of Va neurons, but in this case co-expression with Dac was not observed (Fig. 1B). At stage 16, every abdominal segment expressed Dac/Dimm, and Capa starts to appear in segments A2 to A6. In thoracic segments the expression of Dimm disappeared, and in subsequent stages no Va neurons could be identified (Fig. 1C). At stage 17 and 18 hours after egg laying (18hAEL), the expression pattern mimicked the situation in larval stages, where only three pairs of Capa-expressing neurons co-express Dimm and Dac, and the first abdominal segment, A1 shows positive staining for Dimm/Dac, but lacks the expression of the neuropeptide (Fig. 1E–F).

The temporal appearance and disappearance of Va neuron markers raised interesting questions regarding the fate of the posterior and the anterior Va neurons. Does their absence indicate a specific down-regulation of the respective transcription factors, or is it a result of selective cell death?

### Va neurons are removed from posterior segments by programmed cell death

Central to programmed cell death (PCD) is the highly conserved caspase family of cysteine proteases (Bergmann et al., 1998). During development, PCD can readily be detected using an antibody against the cleaved and active form of caspase-3 (Nicholson et al., 1995). At embryonic stage 16, caspase-3 staining was observed in posterior Va neurons (Fig. 1D, arrows), but never in A1–A4 or any of the thoracic Va neurons. This suggested that, while PCD may not play a role in shaping the segmental pattern of these neurons in the thorax, it does so in the posterior abdominal segments, thereby paralleling the segmental death of dMP2 and MP1 neurons (albeit in the opposite direction) (Miguel-Aliaga and Thor, 2004). The death of posterior Va neurons was further confirmed by making use of the novel Apoliner transgenic reporter for PCD (Bardet et al., 2008). Apoliner is a membrane-RFP to nuclear-GFP fusion protein linked by a caspase-3 cleavage site. In living cells, the Apoliner fusion protein is predominantly localized to the cellular membrane, while in dying cells nuclear-GFP translocates to the nucleus. Segmental death was confirmed by expression of *UAS-Apoliner* from the *dac-Gal4* driver, which revealed PCD of Va neurons in posterior segments (A5–A8) but not in the A2–A4 segments (Fig. 1G).

### Programmed cell death of Va neurons depends upon different RHG genes

**Fig. 2:**
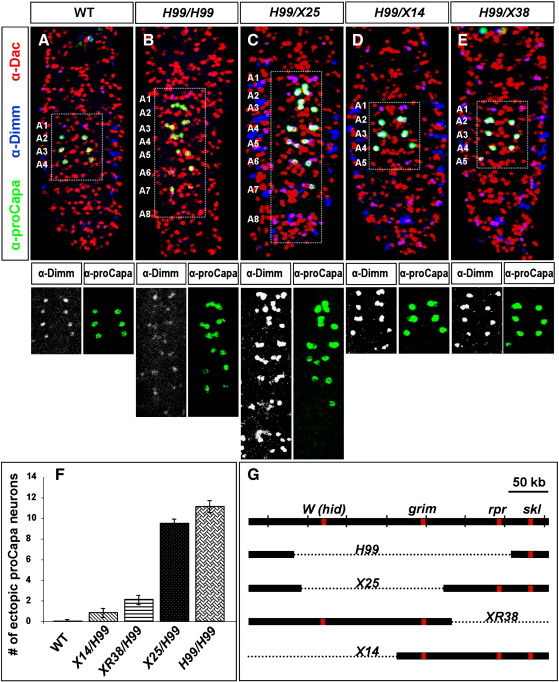
RHG genes are involved in the removal of abdominal Va neurons. (A) In the *Drosophila* VNC, at late embryonic stage (18hAEL), four pairs of abdominal Va neurons express the transcription factors Dac and Dimm (A1–A4), and three of them express the Capa neuropeptides, marked with the proCapa antibody (A2–A4). (B) In late embryos (18hAEL) homozygous for the death activator genes *hid*, *grim*, and *rpr* (*H99/H99*), Va neurons survive in all abdominal segments. (C) *H99/X25* reflects the situation in *H99/H99*, indicating a minor involvement of *rpr* in the PCD of Va neurons. (D) Deficiencies for *hid* alone (*H99/X14*), and (E) *rpr* alone (*H99/XR38*) have a much smaller effect on the survival of posterior Va neurons. (F) Graphical demonstration of the number of ectopic proCapa neurons in the different deletions. Shown are mean values +/− SE (*H99/H99*, *n* = 6*; X25/H99*, *n* = *16; X14/H99*, *n* = 19; *XR38/H99*, *n* = 13; WT, *n* = 27 VNCs). (G) Outline of the different deficiencies in the RHG region.

Important key players in neuronal PCD are the death activators *reaper* (*rpr*), *head involution defectiv*e (*hid*), and *grim*, that each contains a conserved NH_2_-terminal Rpr, Hid, Grim (RHG) motif (Bergmann et al., 2003). The deficiency *Df(3L)H99* (*H99*) removes these genes (Fig. 2G), and embryos homozygous for *H99* display complete absence of PCD (White et al., 1994). Late *H99* embryos (18hAEL) showed a survival of abdominal Va neurons (Fig. 2B), confirming the role PCD plays in shaping the segmental appearance of these peptidergic neurons. Interestingly, additional ectopic Va neurons were present, which were identified by their co-expression of Dac and Dimm and, especially in anterior abdominal segments, also showed positive proCapa staining. These two to three Va neurons in each hemisegment clustered in close proximity to each other, which could indicate that they were siblings, and under normal developmental conditions, cell number control is achieved by PCD. These additional neurons were never visible during normal development, indicating that their death occurs before Dac/Dimm expression starts, suggesting that they probably die directly after they were generated. Towards the posterior end of the VNC, Capa expression declined (A7), and in A8 no Capa could be found, though the presence of the Va neurons could be confirmed via Dac/Dimm. This, together with the fact that Va neurons in A1, the most anterior abdominal segment, never express Capa (Fig. 2A) indicates that factors responsible for the differentiation towards the peptidergic fate of Va neurons are missing in these segments of the VNC. Hence, in addition to PCD, the segmental patterning of Va neurons is guided by other cues.

The thoracic segments T1 to T3 of late embryos, even in *H99* homozygotes, did not show any Va cells positive for either Capa or Dac/Dimm, which parallels the situation in wild type (Fig. 2A–B). This, together with the lack of Caspase-3 staining, favors the idea that PCD is not responsible for the disappearance of thoracic Va neurons.

In order to investigate which of the death activator genes are responsible for the apoptosis of the abdominal Va neurons, smaller deletions were analyzed (Fig. 2). In X14 *hid* is missing, X25 deletes *hid* and *grim* and XR38 deletes the *reaper* gene (Peterson et al., 2002). The single deletions of either *hid* or *reaper* showed a small, but statistically significant effect (Fig. 2F; *P* = 0.01 and *P* < 0.0001, respectively, using Student's *t*-test) on the survival of the Va neurons, when compared to wild type (Fig. 2D–F). By contrast, the combined absence of *hid* and *grim* prevented apoptosis to nearly the same extent as the combined deletion of the three genes in *H99* (Fig. 2C, F; *P* = 0.037). This could indicate a major involvement of *grim* in the initiation of PCD in these neurons, as it is shown in the case of anterior MP1 neurons (Miguel-Aliaga and Thor, 2004). As all the smaller deletions were heterozygous over *H99* a synergistic effect cannot be excluded, though homozygotes for *hid* (*X14/X14*), as well as for *rpr* (*XR38/XR38*) showed no difference (data not shown). However, the possibility that hid and grim act synergistically cannot be excluded since both genes are removed by the X25 deficiency.

### *Abdominal-B* acts in a pro-apoptic role in early-born Va neurons

**Fig. 3:**
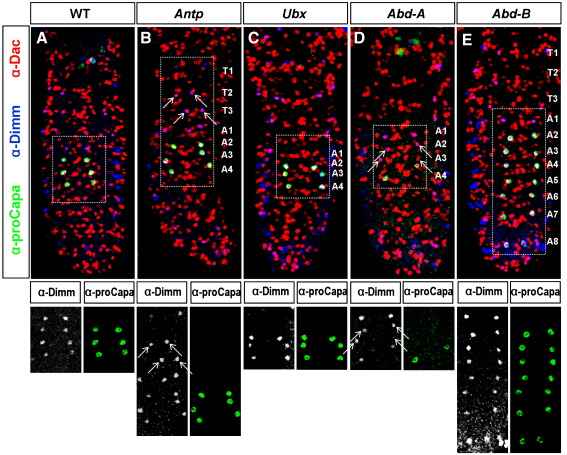
Hox genes differentially affect the developmental progression of Va neurons. (A) In late wild-type embryos (18hAEL), Va neurons are restricted to the anterior abdominal segments (A1–A4). (B) The same embryos of *Antp* mutants (*Antp*^*12*^/*Antp*^*14*^) possess two more pairs of Dac/Dimm-positive Va neurons in the thoracic segments T2 and T3 (arrows). (C) In *Ubx* mutant embryos (*Ubx*^*9.22*^/*Ubx*^*927*^), the Dac/Dimm-positive Va neuron of A1 is missing. (D) When the expression of *abd-A* is missing in late *abd-A*^*P10*^/*abd-A*^*MXI*^ embryos, proCapa expression is either diminished or completely absent (arrows) in Va neurons, while the total number of Va neurons (four pairs) stays the same. (E) In embryos lacking *Abd-B* (*Abd-B*^*M1*^/*Abd-B*^*M2*^) abdominal Va neurons survive from A1–A8, and all, except the one pair in A1, express proCapa.

Hox genes are known to specify the identity of body segments along the anterior–posterior axis in most animals (Hirth et al., 2001; Hirth and Reichert, 1999; Rogulja-Ortmann and Technau, 2008; Sprecher et al., 2004), and several recent studies have demonstrated their involvement in segment-specific neurogenesis (Bello et al., 2003; Berger et al., 2005b; Karlsson et al., 2010). In the *Drosophila* VNC, the Hox genes are represented by *Antennapedia* (*Antp*), *Ultrabithorax* (*Ubx*), *abdominal A* (*abd-A*) and *Abdominal-B* (*Abd-B*), which are expressed in overlapping anterior–posterior domains (Hirth et al., 1998). Previous studies have further demonstrated an involvement of Hox genes in the control of neuronal PCD (Miguel-Aliaga and Thor, 2004; Rogulja-Ortmann et al., 2008). Of particular relevance to the current study is the finding that in dMP2 and MP1 peptidergic neurons, the posteriorly expressed Hox gene *Abd-B* was shown to specifically prevent apoptosis in posterior neurons (Miguel-Aliaga and Thor, 2004). In contrast, the posterior removal of Va neurons by PCD would suggest a pro-apoptotic role for *Abd-B*. This was, indeed, found to be the case: in *Abd-B* mutants we find that posterior abdominal Va neurons (segments A5–A8) survive and expressed Capa (Fig. 3E).

### The more anteriorly expressed Hox genes (*abdominal A*, *Ultrabithorax* and *Antennapedia*) control the specification of Va neurons

While *Abd-B* is important for removal of posterior Va neurons by triggering PCD, it cannot account for the missing thoracic Va neurons, and thus we examined the role of the other Hox genes. We found that *abd-A* mutants displayed reduced Capa expression, although the Va neurons were present, as indicated by the presence of Dac/Dimm expressing cells (Fig. 3D). In *Ubx* mutants, Capa was expressed in the three abdominal segments (A2-A4), but the Dac/Dimmed neuron normally present in A1 was not observed (Fig. 3C). *Antp* mutations on the other hand showed Dac/Dimmed-positive Va neurons in two of the three thoracic segments (T2 and T3), which under normal development are missing at this developmental stage (18hAEL; Fig. 3B). Thus, *Antp*, *Ubx* and *abd-A* are all required for proper segmental appearance of Va neurons from A4 and anteriorly.

### Hox genes play different roles in ensuring proper anteroposterior appearance of Va neurons

**Fig. 4:**
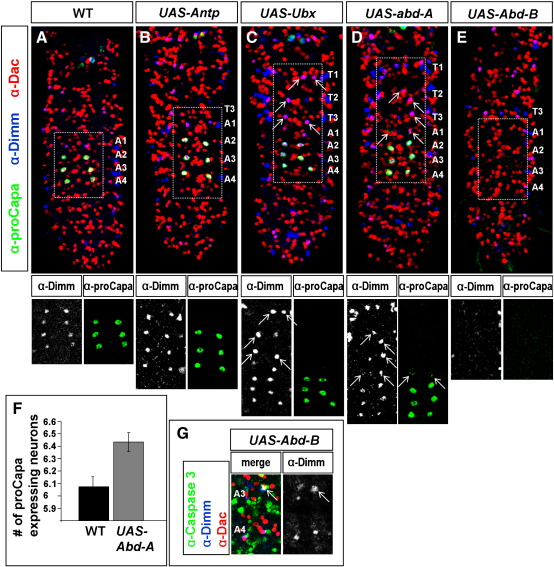
Hox genes influence the survival and differentiation of Va neurons. In wild-type embryos (18hAEL) (A), as well as in embryos with constitutive expression of *Antp* (*UAS-Antp*) using *elav-GAL4* (B), four pairs of Va neurons are found, of which three express proCapa. (C) When *Ubx* is misexpressed from the elav-Gal4 driver (e*lav-Gal4*/*UAS-Ubx*), the thoracic segments T1, T2 and T3 each contains Va neurons, which co-express Dac and Dimm (arrows). (D) Pan-neural expression of *abd-A* (*elav-Gal4*/*UAS-abd-A*) triggers ectopic expression of proCapa (arrows), as well as the maintenance/specification of thoracic Dac/Dimm co-expressing Va neurons (arrows). (E) When *Abd-B* is ectopically expressed from the same driver (*elav-Gal4*/*UAS-Abd-B*), no Va neurons can be found at embryonic stage 18hAEL, and (G) the co-expression of caspase-3 with Dac/Dimm (arrow) in an earlier embryonic stage 15, indicates the involvement of PCD in their disappearance. (F) The number of proCapa expressing Va neurons is significantly higher (*P* = 0.013, Student's *t*-test) in late embryos with constitutive *abd-A* expression (*elav-Gal4*/*UAS-abd-A*) compared to wild type. Shown are mean values +/− SE (WT, *n* = 27; *UAS-abd-A*, *n* = 87 VNCs).

To further investigate which specific roles the different Hox genes play in the patterning of the Va neurons we ectopically expressed them. Our mutant analysis suggested a role for *Abd-B* in removing posterior Va neurons by triggering PCD. In line with this notion, we find that in embryos which expressed *Abd-B* pan-neurally from *elav-Gal4*, no Va neurons could be identified (Fig. 4E–F). This could conceivably be due to the fact that *Abd-B* either triggered PCD of Va neurons, or alternatively blocked Va neuron specification and differentiation. To address this issue, we stained embryos following Abd-B misexpression with Caspase-3 and could identify early apoptotic Va neurons also in the A2–A4 segments. This confirmed the pro-apoptotic role of *Abd-B* on the Va neurons (Fig. 4G).

As demonstrated above, *abd-A* mutants showed reduced Capa expression. In line with this notion, we find that the ectopic expression of *abd-A* leads to a slight increase of ectopic Capa cells, as well as some ectopic Dac/Dimm-positive Va cells in the thoracic region (Fig. 4D). Our *Ubx* mutant analysis revealed an involvement of *Ubx* in the specification of the first pair of Va neurons: the Dac/Dimm expressing Va neuron pair in A1, which does not express Capa. In line with this notion, ectopic expression of *Ubx* resulted in Dac/Dimm-positive Va neurons in all three thoracic segments. However, the abdominal segments A1 to A8 maintained their wild-type expression (Fig. 4C). Finally, our *Antp* mutant analysis revealed a role for this Hox gene in suppressing Va neuron fate in thoracic segments. *Antp* is generally restricted in its capacity to override cues from *Ubx*, *abd-A* and *Abd-B*, and thus, as anticipated, misexpression of *Antp* did not result in any effects upon Va neuron cells in any VNC segment (Fig. 4B).

**Fig. 5:**
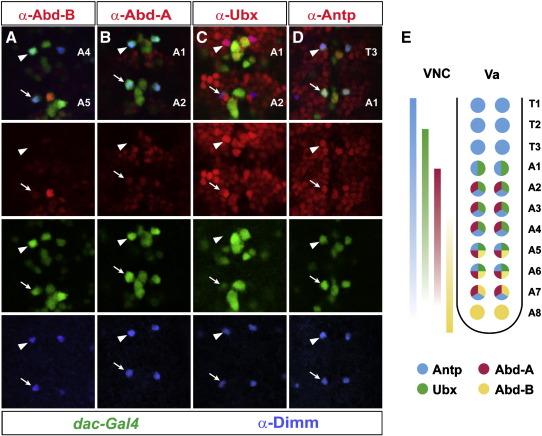
Expression of Abd-B (A), Abd-A (B), Ubx (C) and Antp (D), in the Va neurons at stage 15. Dimm (blue) and dac-Gal4/UAS-eGFP mark the Va neuron in each segment. (A) Abd-B Is expressed in A5-A8 Va neuron (arrow for A5), but not in more anterior Va neurons (arrowhead for A4). (B) Abd-A is expressed in A2-A7 (arrow for A2), but not in A1 Va neurons (arrowhead). (C) Ubx is expressed in A1-A6 Va neurons and is especially strong in A1 (arrowhead), when compared to A2 (arrow). (D) Antp is expressed T1-A7 Va neurons. (E) Summary of Hox protein expression in the Va neurons along the VNC.

To relate these functional data to the actual expression of these four Hox proteins, we mapped their expression in the Va neurons. Starting from the anterior Va neurons, we find that Antp is the only Hox gene expressed at developmental stage 15 in the T1–T3 neurons. Antp is co-expressed together with the other Hox proteins posteriorly, with the exception of A8 which does not express Antp (Fig. 5D). Ubx is expressed From A1 to A6, and Abd-A in A2 to A7 (Fig. 5B–C). It is noteworthy that A1 Va neurons, which depend upon Ubx for specifying the alternate Va neuron subtype, do not express Abd-A (Fig. 5B, arrowhead). Abd-B is found in A5–A8, but importantly its expression does not extend into A4, in line with the mutant analysis for Abd-B (Fig. 5A, arrowhead).

The misexpression of the nerve cord Hox genes thus parallels the mutant analysis and the expression data, with *Abd-B* acting in a pro-apoptic manner, *abd-A* acting to specify Va neurons and *Ubx* acting to specify a modified Va neuron identity. In contrast, *Antp*, although necessary for preventing Va neuron fate in thoracic segments, is not sufficient to block Va neuron determination in abdominal segments.

## Discussion

**Fig. 6:**
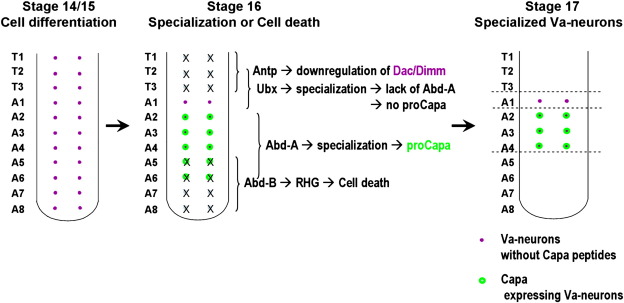
Va cells (depicted in magenta) are characterized by the expression of the transcription factors Dac and Dimm, as well as the neuropeptide Capa (proCapa; green circles). These neurons are initially generated in all VNC segments (T1–A8), and express Dac/Dimm (Dimm in thoracic segments). At stage 16, when in abdominal segments the expression of proCapa begins, Va cells in posterior segments (A5–A8) undergo PCD (crosses). Anterior Va cells (T1–T3) down-regulate Dimm, do not express proCapa and can no longer be detected. However, the PCD analysis indicates that they are not removed by PCD. Va neurons in segment A1 continue to express Dac/Dimm but do not express proCapa. The PCD of posterior Va cells depends upon the RHG genes. Hox genes play several different roles during these processes. In posterior segments, Abd-B acts in a pro-apoptotic manner. In mid-abdominal regions, Abd-A and Ubx are important for the specialization/differentiation of Va neurons. In thoracic segments, Antp appears to oppose Va neuron differentiation.

We have addressed the segment-specific appearance of one peptidergic neuronal subtype, the Capa-expressing Va neurons (Fig. 6). We find that one pair of Va neurons is initially generated in each segment of the VNC. At embryonic stage 14, differentiation begins and the cells commence the expression of the transcription factors Dac and Dimm. Only after this process is initiated, at stage 16, the posteriorly expressed Hox gene *Abd-B* triggers PCD in segments A5 to A8. This PCD involves the RHG motif genes, and our mutant analysis indicates that *grim*, or *grim* and *hid* play the most important roles. As development progresses, the Va neurons in abdominal segments A2–A4 are further specialized under the influence of *abd-A*, which results in expression of the Capa neuropeptide at stage 17. The single pair of Dimm/Dac-expressing Va neurons in the first abdominal segment is present into larval stages, but does not express Capa. These alternate Va neurons depend upon *Ubx* for their Dimm expression, but it is unclear if they differentiate into peptidergic neurons, and if so, which neuropeptide gene they express. In thoracic segments, *Antp* is involved in the down-regulation of Dac and Dimm. These studies unravel a complex interplay of Hox gene input critical for the segment-specific survival and differentiation of the Va neurons and thereby highlight the involvement of Hox genes during the process of shaping the segment-specific structures of the nervous system.

### Hox genes and neuronal subtype specification

Ectopic appearance of Capa expression through ectopic expression of *abd-A* indicates that *abd-A* is an important partner in the combinatorial code of transcription factors necessary for initiating the expression of Capa. The roles of *Ubx* and *Antp* are not as straightforward to assess. *Ubx* showed a participation in the specification of the Va neurons in more anterior segments of the VNC, mainly the thoracic area. Ectopic *Ubx* expression resulted in maintained Dac/Dimm expression in thoracic Va cells into late embryonic stages (18hAEL). Its endogenous role seems to be confined to segment A1, which is characterized by co-expression of Dac/Dimm and a lack of Capa. The role this pair of neurons plays is unknown, as they are not known to express any neuropeptide. The mutant analysis indicates a possible role of *Antp* in the down-regulation of Dac/Dimm in thoracic Va neurons. The ectopic expression of *Antp* however could not override specification signals provided by the other factors (Fig. 4B).

Several studies have identified roles for Hox genes in specifying neuronal subtypes (Dasen and Jessell, 2009). Of particular interest for the current study are previous findings that *Antp* acts at a late stage to specify two other neuropeptide cells; the thoracic *Nplp1* and *FMRFa* neurons of the Apterous (Ap) cluster (Karlsson et al., 2010). Here, *Antp* first acts together with the temporal gene *castor* to activate expression of the *collier* gene, an EBF family member, thus triggering specification of a transient “generic” Ap cluster neurons identity. Subsequently, *Antp* acts in a feedforward manner with *collier* to activate late cell fate determinants, such as *dimm*, and ultimately the *Nplp1* and *FMRFa* neuropeptide genes. Currently, the neuroblast origin of the Va neurons is unclear. Double-labeling with the neuroblast row 5–6 marker GooseberryNeuro indicates that Va neurons originate from a row 5 neuroblast (S. Thor, unpublished observation). As the neuroblast origin of the Va neurons is established, and this lineage mapped, it will be possible to place the generation of Va neurons within a lineage tree. This will furthermore allow identification of the temporal window that generates Va neurons.

### Hox genes and programmed cell death

Programmed cell death plays a critical role in the generation of segmental diversity (Lohmann et al., 2002; Rogulja-Ortmann et al., 2007). Studies in the *Drosophila* embryo have revealed that this can act both at the level of progenitor and postmitotic, even differentiated cells (Bello et al., 2003; Cenci and Gould, 2005; Miguel-Aliaga and Thor, 2004; Rogulja-Ortmann et al., 2008). In progenitors, PCD acts to remove many abdominal neuroblasts after they have completed their lineages and become quiescent (Cenci and Gould, 2005; Prokop et al., 1998). This ensures that as neuroblasts re-enter proliferative states in the larvae, the abdomen has very few quiescent neuroblasts that can enter the cell cycle. Thus, in the adult CNS, the abdomen will end up containing substantially fewer neurons and glia. In postmitotic cells, PCD acts in two apparently different ways: (1) to remove certain postmitotic cells immediately after mitosis (Lundell et al., 2003), or (2) to remove differentiated neurons (Miguel-Aliaga and Thor, 2004; Rogulja-Ortmann et al., 2008). A particularly relevant case to the studies presented is the removal of the peptidergic dMP2 and MP1 neurons. These cells are generated in all VNC segments, extend axons to pioneer critical axon tracts, and subsequently undergo PCD in all segments but the A6–A8 segments (Miguel-Aliaga and Thor, 2004). Strikingly, here *Abd-B* has an anti-apoptotic and promotes peptidergic identity (ADD REF PLOS) role, while in the Va neurons it has a pro-apoptotic role. Moreover, the cell death of both MP1 and Va neurons also depends upon the RHG genes. These results suggest that *Abd-B* acts in an opposing manner, pro- versus anti-apoptotic, by differentially controlling the same PCD pathway in related neurons. An attractive and simple model for this dual role of *Abd-B* would be that MP1 and Va neurons express different regulatory genes, which can act with *Abd-B* to trigger either survival or death. Further studies of PCD in the dMP2, MP1 and Va neurons may help shed light on the molecular genetic mechanisms behind these dual roles of *Abd-B*.
